# Changes in Cooking Characteristics, Structural Properties and Bioactive Components of Wheat Flour Noodles Partially Substituted with Whole-Grain Hulled Tartary Buckwheat Flour

**DOI:** 10.3390/foods13030395

**Published:** 2024-01-25

**Authors:** Mengna Zhang, Zhigang Chen

**Affiliations:** College of Food Science & Technology, Nanjing Agricultural University, Nanjing 210095, China; 2020208005@stu.njau.edu.cn

**Keywords:** whole-grain hulled Tartary buckwheat noodles, cooking characteristics, structural properties, rutin content, starch digestibility

## Abstract

The whole-grain, hulled Tartary buckwheat flour (HTBF) with outstanding bioactive functions was prepared, and the effects of partial substitution ratios (0, 30%, 51% and 70%) of wheat flour with HTBF on the characteristics of TB noodles (TBNs) were investigated, mainly including the cooking characteristics, sensory analysis, internal structure, bioactive components, and in vitro starch digestibility. With an increasing replacement level of HTBF, the water absorption index of the noodles decreased, whereas the cooking loss increased. A sensory analysis indicated that there were no off-flavors in all TBN samples. The scanning electron microscope images presented that the wheat noodles, 30% TBNs and 70% TBNs had dense and uniform cross sections. Meanwhile, the deepest color, V-type complexes, and lowest crystallinity (13.26%) could be observed in the 70% TBNs. A HTBF substitution increased the rutin content and the total phenolic and flavonoid contents in the TBNs, and higher values were found in the 70% TBNs. Furthermore, the lowest rapidly digestible starch content (16%) and highest resistant starch content (66%) were obtained in the 70% TBNs. Results demonstrated that HTBF could be successfully applied to make TBNs, and a 70% substitution level was suggested. This study provides consumers with a good option in the realm of special noodle-type products.

## 1. Introduction

Tartary buckwheat (*Fagopyrum tartaricum* Gaertn; TB), as a kind of pseudocereal, is rich in dietary fiber, B vitamins, minerals and antioxidants (mainly flavonoids) [1,2]. It is grown in mountainous regions like southwest China, northern India and Bhutan. Usually, the nutritional value of TB is much higher than that of common buckwheat (*Fagopyrum esculentum* Moench), especially the content of flavonoids like rutin [3]. Flavonoids have a significant inhibitory effect on chronic diseases caused by oxidative stress, such as cancer, high blood pressure and hyperlipidemia [4,5]. In addition, the content of resistant starch in TB is also high. Gelencsér et al. [6] have found that resistant starch could balance the blood sugar response. TB is viewed as a nutritionally valuable and health-beneficial food material. However, the consumption rate of TB is not very high. The research revealed that the consumption of TB and other miscellaneous food products could effectively prevent and control the occurrence and development of chronic diseases [7]. It was found that the high incidence of chronic diseases such as diabetes, high blood pressure and heart disease, and the decline in the age of onset, were importantly related to the long-term consumption of refined wheat and rice [8]. Thus, expanding the use of this pseudocereal will happen through its application in formulations for mass consumer goods such as TB noodles (TBNs).

Buckwheat noodles, also known as soba in Japanese dishes, have been consumed for centuries in many southeast Asian countries. TB flour (TBF) is commonly mixed with wheat flour (WF) during processing, because a pure TBN is easy to break, and the soup usually becomes turbid during cooking [9]. The protein composition of TB is different from that of wheat, which makes it hard to form an effective gluten network [10,11]. Therefore, TBF cannot be used in large quantities (normally no more than 30%) using conventional thermal treatment and milling techniques [12]. TB has a dense outer layer structure called seed husk, also known as hull, which causes difficulty in grinding and releasing active ingredients and is usually discarded as low-value scrap. It was reported that the main bioactive components in TB, such as phenolic compounds and dietary fiber, were mainly distributed in the buckwheat outer layer structure [11]. Heat fluidization equipment is similar to a high-temperature fluidization bed, which is equipped with a combined infrared and hot-air heating system. It has been reported that heat fluidization could destroy the endosperm cell walls and outer layers of whole-grain highland barley, and significantly enhance free phenolics, β-glucan extractability, antioxidant activity and reducing power, revealing the potential for the development of functional foods [13]. Thus, it is an interesting research topic to focus on innovating and applying modern food processing technology to, to destroy the hull to develop whole-grain, hulled TB (grain with hull) foods like TBNs.

In this study, heat fluidization was applied to dry and puff the TB grains with hull (HTB), and the ultramicronization technique was used to micronize the treated grains and obtain HTB flour (HTBF). The objective of this study was to investigate the effect of different substitution levels of micronized HTBF on the cooking characteristics, sensory analysis, internal structure, bioactive components and in vitro starch digestibility of the noodles, and find an acceptable addition level for HTBF in TBNs to achieve a high nutritional quality.

## 2. Materials and Methods

### 2.1. Materials

HTB and WF (Arawana, multipurpose wheat core flour, protein 11.0%, fat 1.6%, carbohydrates 73.4% and moisture 14.0%) were purchased from the local market (Nanjing, China). HTBF was blended into WF at three substitution levels (30%, 51% and 70%) according to previously reported studies and US Food and Drug Administration (1994) guidelines for making TBNs, and a 100% WF noodle was used as the control group [14,15]. A glucose oxidase/peroxidase (GOPOD) kit was provided by Megazyme International Ireland Ltd. (Bray, Ireland). A plant total phenol (TP) kit, total starch (TS) kit and amyloglucosidase (100 U/mg) were purchased from Beijing Solarbio Science & Technology Co., Ltd. (Beijing, China). A rutin standard solution was bought from J&K Scientific Ltd. (Beijing, China). Pepsin (2400 U/mg) and pancreatin (8 × USP) were purchased from Sigma–Aldrich (St Louis, MO, USA). Methanol, sodium nitrite, aluminum nitrate and sodium hydroxide were provided by Aladdin Bio-Chem Technology Co., Ltd. (Shanghai, China).

### 2.2. Preparation of Whole-Grain HTBF

HTB (5 kg) was added to industrial thermal fluidization equipment (GW-100, Number Times Technology (Huai’an) Co., Ltd., Huai’an, China), and heated at 110 °C, 160 °C, 150 °C, 140 °C and 125 °C for 4 min per stage, respectively, with a superficial air velocity of 3.0 m/s. Afterwards, the thermally-pretreated HTB was micronized using ultramicronization equipment (CW150, Number Times Technology (Huai’an) Co., Ltd., Huai’an, China) and passed through 100 mesh sieves to obtain the whole-grain HTBF (protein content 10%, fat content 3%, moisture 9.59% and total dietary fiber content 40%).

### 2.3. Preparation of TBNs

According to the previously optimized formula in Table 1, WF and HTBF were mixed, and then added to the dough mixer. Salt previously dissolved in the required amount of room-temperature water was slowly added to the dough mixer to make the noodle dough, running for 10 min. The dough was cured for 60 min. A laboratory twin-screw, corotating extruder (Yongkang Wurui Industry and Trade Co., Ltd., Jinhua, China) was used to produce TBNs. The dough was continuously rolled to obtain thin pieces (about 20 mm), which were cut into 20 cm long noodles with a 1.5 mm cutter. Finally, the noodles were subjected to a tunnel dryer (JY-0139, Number Times Technology (Huai’an) Co., Ltd., Huai’an, China) with conditions of 105 °C for 10 min to reduce the final moisture content to 10%.

### 2.4. Cooking Characteristics of TBNs

The cooking characteristics of TBNs, mainly including the optimal cooking time, water absorption and cooking loss, were measured according to the method as described by Bai et al. [14] with some modifications. In brief, 30 strands of noodles with a length of 10 cm each were placed into 750 mL of boiling water. Starting at 6 min, 1 strand of noodle was taken every 15 s and squeezed between two glass slides to observe whether there was a white core. The optimal cooking time was fixed according to the disappearance time of the white inner core. A total of 30 strands of noodles with a length of 10 cm each were weighed and put into 750 mL of boiling water for cooking until the optimal cooking time was fixed. Afterwards, the wet noodles were taken out, and the surface moisture was blotted with blotting paper. The water absorption was the ratio of the mass difference of the noodles after and before cooking to the mass of the noodles before cooking. Then, the remaining water was evaporated to less than 400 mL and cooled down to room temperature. Next, the noodle soup was regulated to 500 mL with deionized water. An aliquot of 100 mL was added to the beaker, evaporated to less than 10 mL and placed in an air oven at 105 °C to a constant weight. The residue was weighed, and the cooking loss was calculated as:

(1) $$\mathrm{Cooking}\;\mathrm{loss}=\frac{5\times Constant\;weight}{Weight\;of\;raw\;noodles\times (1-moisture\;content\;of\;raw\;noodles)}\times 100\%$$

### 2.5. Texture Properties of Cooked TBNs

According to the method used as described by Fu et al. [15] with some modifications, the texture properties were evaluated through a texture profile analysis (TPA) using the TMS-Pro Texture analyzer (TMS-Pro, Food Technology Corporation, Sterling, VA, USA). A set of three strands of cooked noodles were parallelly placed on a flat plate and compressed using a cylinder probe (38 mm in diameter) at a speed of 1.0 mm/s with a trigger force of 0.1 N under a 75% strain. The interval between the two TPA compression cycles was 2 s. The texture properties were calculated as the average of six measurements.

### 2.6. Sensory Evaluation

The evaluation was based on appearance (color and surface appearance), taste, texture and overall acceptability. During the assessment, panel members were instructed to rinse their mouths with water between each sample test. The evaluation criteria were referenced from the National Standard of the People’s Republic of China GB/T 35875-2018 [16] with modifications, and the TBNs were subjected to a percentage-based sensory scoring according to the criteria outlined in Table 2. Regarding the overall acceptability, a score of 60 is considered the acceptable minimum threshold.

### 2.7. Observation of Surface Morphology and Scanning Electron Microscope (SEM)

Images of four noodle samples were collected using a mirrorless interchangeable-lens camera (EOS M50, Canon Corporation, Tokyo, Japan). Noodles were placed on white paper and put into a small photo studio (Deep, Zhejiang Meinuo Photographic Equipment Co., Ltd., Jinhua, China; 400 × 400 × 400 mm).

The stereo microscope (SM) of noodles was measured using the SM instrument (SteREO Discovery.V8, Carl Zeiss Microscopy GmbH, Jena, Germany) and the brief operation was as follows: Firstly, the operating software interface of the stereo microscope was opened, and then the microscope zoom knob was adjusted to a magnification of 6.3. After that, the noodles were placed on the microscope stage. The focusing hand wheel and the light were adjusted until the observed image was clear on the computer monitor. The total magnification was 6.3 × 10 × 1.5.

Four groups of dry noodles were freeze-dried (LGJ-18S, Beijing Songyuan Huaxing Technology Develop Co., Ltd., Beijing, China). The cross sections of noodles were coated with a layer of gold using an ion sputter coater, examined and photographed using an SEM (QUANTA 250 FEG, Thermo Fisher Scientific, Waltham, MA, USA). Micrographs were taken using 500× and 2000× magnifications.

### 2.8. X-ray Diffraction (XRD) Analysis

An XRD study of noodles was performed using a Bruker D2 PHASER X-ray diffractometer (Bruker AXS Inc., Billerica, MA, USA) equipped with Cu radiation at a wavelength of 1.5406 Å. Samples were freeze-dried and milled with a high-throughput tissue grinder (WK-700ML, Nanjing Lisigao Instrument Equipment Co., Ltd., Nanjing, China), passed through an 80-mesh sieve and scanned from 4 to 40° (2θ) at a scanning rate of 2°/min at room temperature. The degree of relative crystallinity in the noodle sample was calculated with MDI-Jade 6.0 software (Materials Data, Inc., Aubrey, TX, USA).

### 2.9. Fourier Transform Infrared Spectroscopy (FTIR)

Briefly, 2 mg of a dry noodle flour sample was weighed and mixed with 100 mg of finely ground KBr to test the spectrum. When scanning, the influence of carbon dioxide and water in the air was automatically deducted, and the spectrum of the sample was deducted from the KBr background. An IR 200 spectrometer (Thermo Fisher Scientific, Madison, WI, USA) was used to collect the IR spectra of different noodle samples, with a measurement range of 4000 to 400 cm^−1^ and 64 scans at a resolution of 4 cm^−1^. Infrared spectroscopy was analyzed using OMINIC 8.2 software for baseline calibration and deconvolution.

### 2.10. Rutin Determination Assay

Dry noodles (1 g) were cut to about 5 mm long and added to the methanol (10 mL) in a 50 mL centrifuge tube and ultrasonically treated (40 °C, 100 W for 60 min). A total of 1.0 mL of the supernatant was taken at 5, 10, 20, 30, 40 and 60 min intervals and was filtered through a 0.45 μm syringe-type organic filter before detection using HPLC (LC-20AD & SPD-M20A, Shimadzu Corporation, Kyoto, Japan). According to the method used as described by Huang et al. [17], HPLC conditions were as follows: column: BDS HYPERSIL C18 (4.6 mm × 150 mm, 5 μm, Thermo Fisher Scientific, Waltham, MA, USA); mobile phase: methanol/water solution (50/50, *v*/*v*); injection volume: 10 µL; column temperature: 35 °C; flow rate: 0.6 mL/min; detection wavelength: 254 nm. The identification and quantification were accomplished by comparing the retention times of peaks in the methanol solution to those of the standard compounds (y = 37420x − 508621, R^2^ = 0.9984).

### 2.11. Total Flavonoid Content (TFC) and Total Phenolic Content (TPC) Assay

TFC was determined using the method described by Guo et al. [18] with little modification. A total of 0.1 g of the dry noodle was mixed with 2.0 mL of absolute methanol, and the mixture was subjected to ultrasound extraction at a frequency of 60 Hz for 10 min. The extracted solution was centrifuged at 3500 rpm for 12 min, and the supernatant was collected. The process was repeated three times in total. The extracting solution (3 × 2.0 mL) was combined and dried using a vacuum evaporator. The dried extractive was dissolved in 2.5 mL of 80% methanol for further analysis. A total of 50 µL; of sample was reacted with 100 µL; of 5% sodium nitrite for 6 min, and then, 100 µL; of 10% aluminum nitrate was added to stand for another 6 min. After that, 1.0 mL of 4% sodium hydroxide was added, and the reaction solution was adjusted to 2.5 mL with 1250 µL; of deionized water. After reacting for 15 min, the absorbance was measured at 510 nm and compared to that of rutin standards (y = 0.1549x − 0.0015, R^2^ = 0.9997).

TPC was determined using the Solarbio plant total phenol content assay kit according to the instructions of the manufacturer. TPC was expressed in mg of gallic acid equivalents (GAE) per g of sample.

### 2.12. In Vitro Starch Digestibility

The in vitro starch digestibility of TBNs was tested according to the methods described by Sun et al. [19]. Cooked noodles (2.5 g) at the optimal cooking time were cut to 1–3 mm long and put in a 100 mL conical flask with 30 mL of water. Then, the conical flask was placed in a 37 °C water bath for 10 min of stirring. The sample liquid was incubated for 30 min after the pH was adjusted to 2.5 using 1 mol/L aqueous HCl and 0.8 mL of 2% pepsin solution was added. Afterwards, 0.5 mL of the aliquot was taken out and immediately mixed with 2 mL of absolute ethanol in a centrifuge tube. Then, 1 mol/L NaHCO_3_ was used to adjust the pH to 6.2, and 0.5 mL of 3300 U/mL amyloglucosidase was added before the addition of 5 mL of 5% pancreatin. The final digestion volume was adjusted by adding 15 mL of distilled water. A total of 0.5 mL of the aliquots was taken at 20, 60, 90, 120 and 180 min intervals and immediately mixed with 2 mL of absolute ethanol in a centrifuge tube. A total of 0.1 mL of supernatant was taken to measure the glucose content with a d-glucose assay kit. The starch amount was calculated by multiplying glucose levels by 0.9. The rate of starch digestion was expressed as the percentage of total starch hydrolyzed at different times. Rapidly digested starch (RDS), slowly digested starch (SDS) and resistant starch (RS) were calculated according to the methods described by Sun et al. [19].

### 2.13. Statistical Analysis

Results were expressed as the mean ± standard deviation (SD). A statistical analysis of the data was performed using SPSS (version 21.0, SPSS Inc., Chicago, IL, USA). Differences in means were determined using Duncan’s multiple range test, and *p* < 0.05 was deemed statistically significant throughout the study. OriginPro 2021 (Originlab, Northampton, MA, USA) was used for graphing. All measurements were performed in triplicate unless specifically described.

## 3. Results and Discussion

### 3.1. Cooking Characteristics and Texture Properties of Noodles

**Figure 1:**
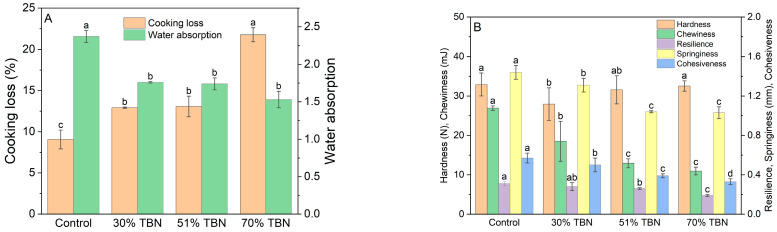
Cooking loss and water absorption (**A**) and texture properties (**B**) of different noodle samples. Different letters (a–d) in the same index indicate significant difference at *p* < 0.05.

The cooking characteristics of the wheat noodles and TBNs are shown in Figure 1. With an increasing amount of HTBF, the water absorption (Figure 1A) of the noodles decreased, and the differences between the wheat noodles (2.37) and TBNs were significant (*p* < 0.05). Nevertheless, the water absorptions of the 30% TBNs, 51% TBNs and 70% TBNs (1.76, 1.74 and 1.53, respectively) were similar, showing no significant differences. Wheat noodles contained much more gluten than that of the TBNs, which could absorb a greater quantity of water [20,21]. In addition, the content of starch, damaged starch and soluble fiber components (especially in BF) may also affect the water absorption of cooked noodles. Conversely, the cooking loss of cooked noodles increased with the increasing substitution level of HTBF (Figure 1A). The lowest cooking loss of 9.06% occurred with wheat noodles and the highest one of 21.76% was with 70% TBNs. The cooking losses in the 30% and 51% TBNs (12.92% and 13.07%, respectively) showed no significant differences, which were much lower than that in the 70% TBNs (21.76%). It was reported that the differences in the water absorption and cooking loss were decided by the structural compactness of the noodles, the extent of gelatinization and the strength of the retrograded starch network surrounding the gelatinized starch [2,22]. Moreover, it was found that compared with fresh buckwheat noodles, dried buckwheat noodles exhibited a higher cooking loss, possibly interpreting the higher cooking loss obtained in this study [23]. The cooking loss of TBNs was higher than that of wheat noodles, because HTBF contained a relatively higher content of fiber, lipids and other components and a lower gluten content, which destroyed the integrity of the gluten–starch network and led to the soluble substances easily leaching out [15].

The eating qualities of the wheat noodles and TBNs were measured quantitatively using a texture analyzer under a TPA mode mainly including the hardness, resilience, cohesiveness, springiness and chewiness (Figure 1B). These indicators are some of the major attributes that consumers are concerned about. Hardness represents the anticompression ability of the cooked noodles, which is defined as the peak force at the first compression [14]. As the substitution level of HTBF increased, the hardness of the noodles first decreased and then increased, with the highest value of 32.92 N in the control group and the second highest value of 32.53 N for the 70% TBNs. The hardness of the 30% TBNs was relatively lower than that of other groups, while it revealed no significant difference with the 51% TBNs. The results were mainly attributed to the integrated and rigid gluten–starch network formed in wheat noodles after the cooking process, which was also consistent with the results of the cooking loss and water absorption. Although the fiber increased and gluten decreased with the HTBF substitution level increasing, the hardness enhanced when the HTBF level further rose to 51% and 70%, as the increased amylose content provided the gels with a more intensive and stronger network and made the noodles have a harder texture [15]. Compared to control group, the hardness of the 51% and 70% TBNs showed no significant difference (*p* < 0.05), which indicated that TBNs displayed a preferable hardness despite the addition of HTBF. As the replacement rate of HTBF increased from 0 to 70%, the resilience, cohesiveness, springiness and chewiness both decreased. Lesser influences on the resilience, cohesiveness and springiness were exhibited, but there was a greater impact on chewiness. Based on the analysis of these five indicators, it was revealed that the addition of HTBF could affect the texture properties. The TPA results were attributed to the fact that more fiber and less gluten were comprised in TBNs compared to wheat noodles, leading to a relatively less compact gluten–starch network in TBNs, which was also reflected by the cooking loss results from a sideways perspective.

### 3.2. Sensory Analysis

**Figure 2:**
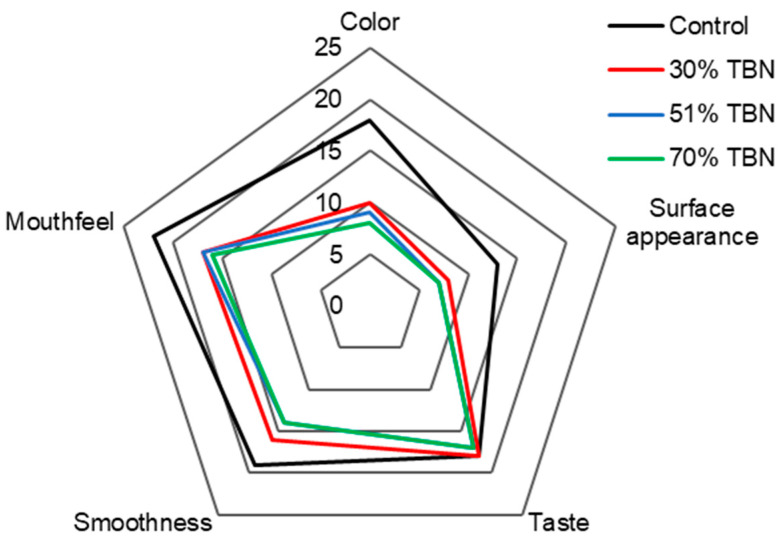
Sensory scores of the control and TBN samples.

The sensory evaluation data recorded in Figure 2 exhibited the appearance (color and surface appearance), taste, texture and overall acceptability of different noodle samples. The taste scores of 30%, 51% and 70% TBNs displayed no obvious differences compared to the control wheat noodles. There were no off-flavors in TBNs, and all TBN samples had a pure and authentic flavor with a cereal aroma, which indicated that the TBNs made of HTBF and WF satisfied the taste demands of the consumers. The scores for surface appearance, smoothness and mouthfeel decreased as the substitution level of HTBF increased, whereas the differences between the TBNs were slight. The color was the most obvious distinction between the wheat noodles and TBNs due to the dark hull of TB, mainly resulting in the overall scores of TBNs being lower than the control. It was reported that with the increase of TBF levels in noodles, the color of the dough sheet became darker [2]. If the score for overall acceptability is above 60, the noodle product is considered acceptable. The overall sensory scores of the control, 30% TBNs, 51% TBNs and 70% TBNs (90, 69, 64 and 62, respectively) exceeded 60, indicating that TBNs with different levels of HTBF replacement were generally acceptable.

### 3.3. Surface Morphology and SEM

**Figure 3:**
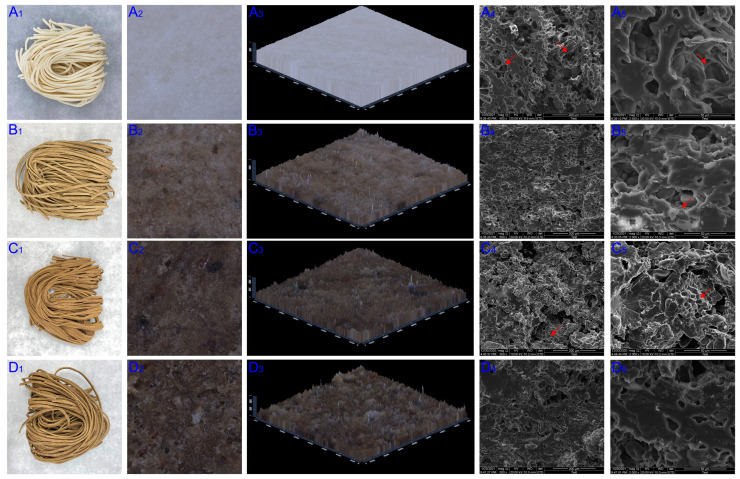
Images from SM and SEM of different noodle samples. The (**A**–**D**) represent the control, 30% TBNs, 51% TBNs and 70% TBNs, respectively; the subscripts 1–5 represent the images of noodles, surface images from SM, 2.5D images from SM, ×500 magnification of SEM, ×2000 magnification of SEM, respectively.

The surface and micromorphology of control and TBN samples were shown with SM and SEM images (Figure 3), which can reveal the appearance, the smoothness level of surface and the gluten–starch network formed within noodles. The images showed that TBNs exhibited a regular shape and with the substitution level increasing, the color became deeper, which could also be seen with the SM. An SM is a kind of stereoscopic microscope with a positive image, which is widely used in material macroscopic surface observation, failure analysis, fracture analysis, etc. As depicted in Figure 3A_2_–D_3_, the surfaces of the noodles got coarser as the addition proportion of HTBF increased, whereas the smoothness levels of the 30% and 51% TBNs were similar except that the color of the 51% TBNs was much deeper. It was clearly observed from Figure 3D_2_–D_3_ that there was a rough and uneven region existing on the 70% TBN surface, and the 2.5 D image was particularly clear.

Large or small starch particles dissociating or adhering to the gluten network were observed in the internal structure of the noodles from the SEM images (Figure 3A_4_–D_5_). The filamentous gluten network was filled with starch granules in the control group (Figure 3A_4_–A_5_). After the WF was replaced with HTBF, the gluten content decreased, and consequently, the gluten network became slightly less obvious, leading to some starch granules being exposed. From Figure 3C_4_–C_5_, some starch granules with different appearances could be clearly observed in the 51% TBNs, which were polygonal, irregular and smaller than wheat starch granules, namely, TB starch granules. The continuity of the starch–gluten network was destroyed to a certain extent with the increasing amount of HTBF, and some holes appeared due to the great amount of fiber contained. However, it is worth noting that the internal structure of the 70% TBNs was more continuous than that of the 51% TBNs, which may be attributed to the enhanced content of amylose.

### 3.4. XRD Analysis

**Figure 4:**
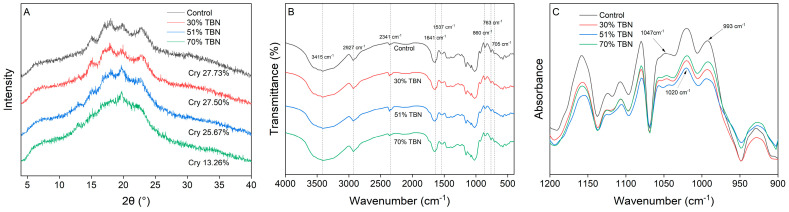
XRD patterns (**A**), FTIR spectra (**B**) and deconvolved IR spectra between 1200 and 900 cm^−1^ (**C**) of different noodle samples.

Figure 4A shows the XRD patterns of the wheat noodles and TBNs, which could be applied to determine the starch crystal structure and crystallinity. The long-range ordered structure reflected through the XRD of the matrices differed depending on the substitution level of HTBF. The wheat noodle flour exhibited strong diffraction peaks at about 2θ = 15°, 17°, 18° and 23°, respectively. Among them, the peaks around 17° and 18° were linked double peaks. Additionally, the wheat noodle flour showed a typical A-type crystal starch pattern (around 2θ = 20°), which was the same as the other seed starches of cereal crops, such as rice starch, corn starch and so on [24]. With an increase in HTBF levels, the shape of the XRD pattern changed obviously. The peaks at about 2θ = 15° and 23° got progressively weaker and broader, and the peaks at 17° and 18° merged into one peak and became weaker until disappeared, while the peaks at 13° and 20° became more and more evident, indicating that the proportion of the V-type crystal peak increased, and the V-type became predominant. The peaks at 2θ = 13° and 20° were generally generated by amylose and lipid [19]. Because the HTBF contained more amylose, it formed more amylose–lipid complexes and enhanced the peak at about 13°and 20°, as obviously seen in the XRD pattern of the 70% TBNs.

However, Cheetham and Tao [24] reported that the degree of crystallinity was inversely proportional to the amylose content. Although peaks of the amylose–lipid complex at 13° and 20° became evident gradually, low and broad dispersion peaks strengthened, transiting from crystal to more amorphous structure, indicating that the long-range ordered structure was destroyed to some extent, which was also reflected by the decreased crystallinity of the TBNs. The control group had the highest crystallinity (27.73%), similar to that of the 30% TBNs, while the 70% TBNs had the lowest (13.26%).

### 3.5. FTIR Analysis

The FTIR patterns of different noodle samples are shown in Figure 4B, and the functional groups and chemical environments are analyzed from 4000–400 cm^−1^. According to the previous literature, the characteristics of several key absorbance bands were interpreted [25,26,27]. A broad peak of 3415 cm^−1^ was ascribed to the O–H stretching vibration of the glucose unit due to hydration of the roux paste [28]. Additionally, there was almost no variation in the peak position of the –OH stretching vibration, which represented negligible changes in the energy between the O–H bonds. The absorption bands at 2927 cm^−1^ and 2341 cm^−1^ were attributed to the asymmetric stretching of –CH_2_ and –CH, respectively, which were involved in the ring methane hydrogen atoms. The 1641 cm^−1^ band in the spectra reflected a C=O stretch in proteins, which was ascribed to the amide I peak. In addition, the 1641 cm^−1^ band represented intra- and inter-molecular hydrogen bonds owing to the adsorbed water. The peak at 1537 cm^−1^ was ascribed to the amide II peak, which reflected the N–H bend and C–N stretch in proteins. The characteristic peaks of the skeletal modes of the pyranose ring in the starches were mainly observed at 860 cm^−1^, 763 cm^−1^ and 705 cm^−1^.

Moreover, the characteristic bands between 1200 and 900 cm^−1^ were further deconvolved to compare the short-range ordered structures of different noodle samples, and are presented in Figure 4C. Studies have shown that the intensities of the absorption peaks at 1047 cm^−1^, 1020 cm^−1^ and 993 cm^−1^ in the infrared spectrum of starch characterize the content of the crystalline and amorphous regions in starch samples, respectively [29,30], so R_1047/1020_ and R_1020/993_ could be used to evaluate the relative content of short-range ordered structures in starch. From Table 3, the R_1047/1020_ of different noodle samples showed no significant difference, which indicated that there were no obvious changes in the double helix structure content of starch with the substitution ratio of HTBF improving, and TBNs could still maintain a relatively ordered short-range structure. The R_1020/993_ of these four samples had a slight increase with the increasing amount of HTBF, and there was no significant difference between control, 30% TBNs and 51% TBNs. This result illustrated that the interaction between the starch and water was enhanced in TBNs. Capron et al. [31] found that the R_1020/993_ had a good correlation with the crystallinity measured using XRD, and the increasing degree of R_1020/993_ can reflect the loss of crystallinity, which was consistent with the results of XRD.

### 3.6. Content of Bioactive Compounds

**Figure 5:**
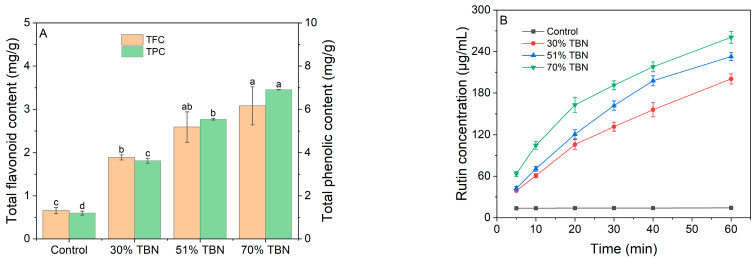
The TFC and TPC (**A**) and rutin concentration changes over time (**B**) of different noodle samples. Different letters (a–d) in the same index indicate significant difference at *p* < 0.05.

The TFC and TPC of noodles with different substitution ratios of HTBF are presented in Figure 5A. It is well known that the hull of TB is sufficient in flavonoids and phenols, which have high health-promoting activities, such as reducing the risk of cardiovascular diseases, strengthening the immune system and antioxidation effects [18]. That was one reason why we tried to retain the hull of the grain to obtain the whole-grain HTBF for producing the noodles. Hence, the TFC and TPC of the noodle samples significantly increased as the proportions of HTBF increased. The 70% TBNs contained the highest contents of TFC and TPC, which were 3.08 mg/g and 6.91 mg/g, respectively.

It can be seen from Figure 5B that the rutin concentration of wheat noodles shows little change, and the content is much lower than that of the other three groups, while the rutin concentration of the TBNs increases rapidly and then slowly with the increase in extraction time. Furthermore, the concentration of rutin at each time point also increased with the addition of HTBF. It was clearly seen that the rutin concentration of the 70% TBNs was higher than that of other samples at each point, and its concentration at the first 20 min (162.94 µg/mL) was notably higher than that of the 30% TBNs and 51% TBNs (105.85 and 120.70 µg/mL, respectively). The final concentrations of rutin at an extraction time of 60 min in the control, 30% TBNs, 51% TBNs and 70% TBNs were 14.20, 200.48, 232.88 and 260.69 µg/mL, respectively. The application of HTBF to the production of TB noodles can greatly improve the nutritional value of TBNs. It may be used as a meal replacement for diabetics.

### 3.7. In Vitro Starch Digestibility

**Figure 6:**
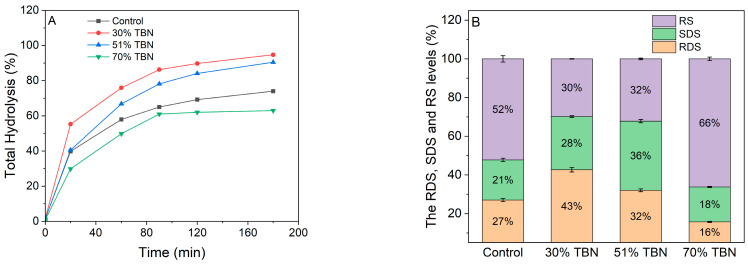
The in vitro hydrolysis (**A**) and the RDS, SDS and RS levels (**B**) of different noodle samples.

The in vitro hydrolysis curves are usually applied to simulate in vivo starch digestion and can be used to calculate the RDS, SDS and RS. The starch hydrolysis curves of noodle samples with different substitution levels of HTBF are presented in Figure 6A. It can be observed that the starch hydrolysis rates of all tested samples and white bread increased rapidly in the first 90 min, especially in the first 20 min, where the starch hydrolysis rate was the fastest. In the last 90 min, it showed a slow growth trend. Among these four samples, the 30% TBNs exhibited the highest hydrolysis ratio, while the 70% TBNs presented the lowest. Moreover, as shown in Figure 6B, the RS content improved and the RDS reduced dramatically when the addition amount of HTBF was 70%, which were 66% and 16%, respectively. The 70% TBNs contained a much higher amylose and dietary fiber content, which can improve the potential sources of RS2, RS3 and RS5 [32,33]. In particular, the amylose–lipid complexes were proposed as RS5 in the 70% TBNs, which were proved to be visibly enhanced through XRD analysis. Therefore, when the amount of HTBF reached 70%, the hydrolysis ratio of starch can be greatly reduced, and the content of RS can be increased.

## 4. Conclusions

In this study, the cooking characteristics, sensory analysis, structural properties, bioactive components and in vitro starch digestibility of TBNs were deeply affected by the whole-grain HTBF with gluten-free properties. The water absorption index of the noodles decreased with the increase in replacement levels of HTBF, but the cooking loss showed the adverse phenomenon. The sensory analysis results showed that there were no off-flavors in TBNs and all TBN samples had a pure and authentic flavor with a cereal aroma. The XRD and FTIR results showed that HTBF enrichment influenced the long-range and short-range ordered structures. Additionally, an HTBF substitution increased the rutin content, TPC as well as the TFC, and higher values were found in the 70% TBNs. Finally, the in vitro digestibility test indicated that RS significantly increased as the substitution level of HTBF rose to 70%. The results demonstrated that higher substitution levels of HTBF were acceptable for producing TBNs. However, there is a need for further comparison of the effects of nontreated HTBF and heat-fluidized HTBF as ingredients in noodles. The 70% TBNs had better nutritional characteristics and a higher RS content, which probably could be used as supplementary food for diabetic patients. In the future, its hypoglycemic activity and effect on the gut microbiota of diabetics will be further studied.

## Figures and Tables

**Table 1:** Formulas of noodles.

Noodles	Addition Amount			
	Wheat Flour g/100 g	Salt g/100 g	Water/mL	Buckwheat Flour g/100 g
Control	100	1.5	30.0	0
30% TBNs	70	1.5	36.0	30
51% TBNs	49	1.5	44.0	51
70% TBNs	30	1.5	47.5	70

**Table 2:** Sensory evaluation criteria for TBNs.

Evaluation Index	Scoring Criteria	Score (Points)
Color (20 points)	Bright white or bright yellow noodles, uniform color and glossy	16–20
	Noodles with average or slightly dark brightness	8–15
	Noodles with dull color and poor brightness	2–7
Surface appearance (15 points)	Smooth surface with a translucent appearance	12–15
	Relatively smooth surface with less noticeable translucent appearance	8–11
	Rough and uneven surface without translucent appearance	3–7
Taste (20 points)	Pure and authentic flavor with a sweet cereal aroma	17–20
	No off-flavors or a mild, sweet cereal aroma	12–16
	Unpleasant or off-putting odor	7–11
Smoothness (20 points)	Smooth and nonsticky texture	17–20
	Relatively smooth with slight stickiness	13–16
	Not smooth and sticky	8–12
Mouthfeel (25 points)	Moderate firmness with chewiness and elasticity	21–25
	Slightly soft or firm with less noticeable chewiness and slightly weak elasticity	16–20
	Very soft or very firm with no chewiness and poor elasticity	10–15

**Table 3:** FTIR deconvolution results of different noodle samples.

Samples	Short-Range Ordered Structure	
	R_1047/1020_	R_1020/993_
Control	0.75 ± 0.02 ^a^	1.11 ± 0.01 ^b^
30% TBNs	0.75 ± 0.01 ^a^	1.11 ± 0.04 ^b^
51% TBNs	0.75 ± 0.00 ^a^	1.20 ± 0.03 ^ab^
70% TBNs	0.74 ± 0.02 ^a^	1.22 ± 0.09 ^a^

Note: The different letters in the same column indicated a significant level at *p* < 0.05.

## Data Availability

The original contributions presented in the study are included in the article, further inquiries can be directed to the corresponding author.
